# High Levels of *N*-Palmitoylethanolamide and *N*-Stearoylethanolamide in Microdialysate Samples from Myalgic Trapezius Muscle in Women

**DOI:** 10.1371/journal.pone.0027257

**Published:** 2011-11-18

**Authors:** Nazdar Ghafouri, Bijar Ghafouri, Britt Larsson, Maria V. Turkina, Linn Karlsson, Christopher J. Fowler, Björn Gerdle

**Affiliations:** 1 Rehabilitation Medicine, Faculty of Health Sciences, Linköping University, and Pain and Rehabilitation Centre, County Council of Östergötland, Linköping, Sweden; 2 Department of Pharmacology and Clinical Neuroscience, Umeå University, Umeå, Sweden; 3 Occupational and Environmental medicine, Department of Clinical and Experimental Medicine, Faculty of Health Sciences, Linköping University, and Department of Occupational and Environmental Medicine, County Council of Östergötland, Linköping, Sweden; 4 Cell Biology, Faculty of Health Sciences, Linköping University, Linköping, Sweden; Sapienza University of Rome, Italy

## Abstract

**Background:**

*N*-acylethanolamines (NAEs) are endogenous compounds that regulate inflammation and pain. These include the cannabinoid ligand anandamide (AEA) and the peroxisome proliferator-activated receptor-α ligand palmitoylethanolamide (PEA). Little is known as to the levels of NAEs in pain states in human, particularly in the skeletal muscle. The aim of this study was to investigate the levels of these lipid mediators in muscle dialysate from women with chronic neck-/shoulder pain compared to healthy controls.

**Methods:**

Eleven women with chronic neck-/shoulder pain and eleven healthy women participated in this study. All participants went through microdialysis procedures in the trapezius muscle. Muscle dialysate samples were collected during four hours and analysed by nano liquid chromatography tandem mass spectrometry (nLC-MS/MS).

**Results:**

We were able to detect AEA, PEA, *N*-stearoylethanolamine (SEA) and 2-arachidonoylglycerol (2-AG) in a single chromatographic run. Of the NAEs studied, PEA and SEA were clearly detectable in the muscle microdialysate samples. The muscle dialysate levels of PEA and SEA were significantly higher in myalgic subjects compared to healthy controls.

**Conclusion:**

This study demonstrates that microdialysis in combination with mass spectrometry can be used for analysing NAE's in human muscle tissue regularly over time. Furthermore the significant group differences in the concentration of PEA and SEA in this study might fill an important gap in our knowledge of mechanisms in chronic myalgia in humans. In the long run this expanded understanding of nociceptive and anitinociceptive processes in the muscle may provide a base for ameliorating treatment and rehabilitation of pain.

## Introduction

Chronic pain is associated with disability, low quality of life and substantial socioeconomic costs. Common chronic pain conditions are localized neck-shoulder pain including trapezius myalgia, which has a prevalence of 10–20% in the community [1]. A bio-psycho-social model [2], [3] is preferred in clinical management of chronic pain since a complex blend of factors - neurobiological, psychological, coping styles, and contextual factors - contributes to the development and maintenance of chronic pain [4], [5], [6], [7], [8]. There are several indications that central alterations in nociceptive processing can be driven by peripheral tissue alterations [9] and peripheral nociceptive input [10], [11] also in the chronic stage of a pain condition. However, the understanding of mechanisms, including the peripheral balances between nociceptive and anitinociceptive processes, behind chronic myalgia is incomplete. Muscle nociception is activated by stimulation of free nerve endings of group III (Aδ) and IV afferent (C) fibers. Based on animal experiments and infusion of different substances into human muscles it is known that nociceptors respond to single or combinations of noxious stimuli: mechanical, temperature, and chemical (i.e., algesics such as serotonin, bradykinin, glutamate and substance P) [12]. The sensitivity of the nociceptors can be increased by endogenous substances [12].

The microdialysis technique offers a well-established *in vivo* method to study nociceptive and metabolic mechanisms [13], [14]. The trapezius muscle can be used as a human model muscle of chronic myalgia both due to its clinical importance and to its accessibility for invasive investigations. Investigating chronic trapezius myalgia using microdialysis techniques, we and others have reported significantly increased interstitial levels of different algesics indicating activated peripheral nociceptive processes [15], [16], [17].

Whilst the microdialysis studies of myalgic muscles so far undertaken implicate changes in algesic signalling as a key event, little is known about changes in the pain-inhibitory signalling molecules. One interesting group of such molecules are the *N*-acylethanolamines (NAEs), a family of endogenous lipid mediators that have a diversity of actions including the regulation of inflammation and pain [18]. Among the NAEs are *N*-palmitoylethanolamine (PEA), *N*-stearoylethanolamine (SEA), *N*-oleoylethanolamine (OEA) and *N*-arachidonoylethanolamine (anandamide, AEA). The most well-studied of the NAEs is AEA, which interacts with cannabinoid receptors and at higher concentrations other targets including transient receptor potential (vanilloid-1) receptors, and has been shown to have anti-nociceptive actions in a number of animal models of pain [19]. However, in most tissues, the concentration of AEA is much lower than of PEA, which has been shown to have both anti-inflammatory and anti-nociceptive effects in several pain models [20], [21], [22], [23], [24], possibly due to its ability to interact with peroxisome proliferator-activated receptor α. OEA is also a ligand for this receptor, but has mainly been investigated with respect to feeding behaviour [25], while much less is known about the *in vivo* functions of the other NAEs.

In view of the potential role of NAEs in the control of pain, the aim of this study was to investigate the levels of NAEs in human muscle microdialysate using a high sensitive liquid chromatography tandem mass spectrometry (nLC-MS/MS) method and to utilise it to determine whether NAE levels are altered in patients with myalgia.

## Materials and Methods

### Study design and procedures

Participants with and without chronic neck-shoulder pain were recruited via advertisements in the local daily newspaper. Those who responded received an invitation letter with information about the study. A self-reported pain questionnaire together with a structured telephone interview was used for primary screening in order to assess eligibility. Eligible patients were invited for a standardized clinical neck and shoulder examination in order to confirm inclusion and exclusion criteria. Consecutive enrolment was applied and the time between clinical examination and microdialysis examination of the trapezius muscle was one to four weeks.

### Participants

#### Subjects with chronic pain

Eleven women with chronic pain in neck/shoulder area participated in the study. Inclusion criteria were female sex, age range 20–55 years, and pain in the neck-/shoulder area that had lasted more than six months. Exclusion criteria were generalized pain such as fibromyalgia, upper extremity bursitis, tendonitis, capsulitis, postoperative conditions in the neck/shoulder area, prior neck trauma, disorder of the spine, neurological disease, rheumatoid arthritis or any other systemic diseases, metabolic disease, malignancy, severe psychiatric illness, pregnancy, BMI>35, and difficulties understanding the Swedish language. Median age and median body mass index (BMI) noted in the records was 45 years and 23.2 kg/m^2^, respectively (**Table 1**).

**Table 1:** Age, anthropometric data and pain duration in the two groups (median, maximum and minimum values).

Variables	Group						Statistics
	Controls			Chronic Pain			
	Median	Minimum	Maximum	Median	Minimum	Maximum	p-value
Age (years)	41	26	54	45	26	55	0.576
Height (cm)	169	155	177	168	154	172	0.449
Weight (kg)	68	55	98	61	57	79	0.470
BMI (kg/m^2^)	24,0	21,9	31,2	23,2	19,9	29,3	0.375
Pain duration (months)	NA	NA	NA	51	9	252	NA

Furthest to the right is given the results of the statistical analyses.

NA = not applicable.

#### Pain-free subjects

Eleven pain-free women comprised a comparison group. Inclusion criteria were female sex, age range 20–55 years and pain-free. The exclusion criteria were as above, and additionally pain that had lasted more than seven days during the past 12 months. Median age and median BMI was 41 years and 24.0 kg/m^2^, respectively (**Table 1**).

No significant differences between groups existed with respect to age and anthropometric variables (**Table 1**).

Anthropometric data together with age and pain duration are reported in **table 1** for the two groups.

### Ethical approval of the study

After receiving verbal and written information about the study, all subjects signed a consent form that was in accordance with the Declaration of Helsinki. The study was granted ethical clearance by the Linköping University Ethics Committee (Dnr: M10-08).

## Methods

### Self-reported pain questionnaire

The Nordic Ministry Council Questionnaire (NMCQ) was used [26]. This is a well-established self-reported pain questionnaire allowing assessment of low back, neck, shoulder and general complaints. It includes symptoms (ache, pain, and discomfort) arising from nine well defined anatomical regions over the past 12 months and the previous seven days.

### Standardized clinical neck and shoulder examination

In order to confirm eligibility for inclusion in the study, all participants were examined by a standardized and validated clinical examination according to Ohlsson et al. [27]. The examination includes questions on pain, tiredness and stiffness on the day of examination, as well as physical tests including; range of motion and tightness of muscles, pain threshold and sensitivity, muscle strength and palpation of tender points. Patients with trapezius myalgia that were included in the study had neck pain at the examination day, tightness of the trapezius muscle, i.e., a feeling of stiffness in the descending region of the trapezius muscle reported by the subject at examination of lateral flexion of the head, and palpable tender parts in the trapezius muscle. The range of motion of the cervical columna was normal or slightly decreased.

### The microdialysis technique

The technique of microdialysis enables the monitoring of different substances in the extracellular environment and is now widely used for sampling and quantitating neurotransmitters, neuropeptides, and other substances in the brain and periphery. Briefly a microdialysis system consists of the microdialysis pump, the microdialysis catheter and a microvial in which the sample is collected. During the process of microdialysis the catheter is inserted in the tissue of interest and is perfused with a solution (termed perfusate) that resembles the interstitial fluid. Substances diffuse into or out of the perfusion fluid in a concentration-dependent manner and dialysate samples can be collected [28].

### Experimental procedure

The participants were asked not to take non-steroidal anti-inflammatory drugs for the last seven days and/or paracetamol medication for the last 12 hours before the experiment. In addition, they were asked not to consume coffee and/or tea and cigarettes and/or nicotine agents for the last eight hours before the experiment. They were also instructed not to perform any shoulder or neck-straining exercises for the last 48 hours before the study, except for ordinary daily work and/or leisure activities.

The participants reported to the laboratory in the morning. The examiner went through a checklist in order to assess whether or not participants had followed the instructions, and their current health status as well as current medication was confirmed. The most painful side (subjects with pain) or the dominant side (pain free subjects) of the trapezius muscle was used for microdialysis procedures. To guide the placement of catheters, ultrasonographic measurements of the trapezius muscle area were conducted. The B-scan application was used measuring skin/fat/muscle thickness of the trapezius muscle area with a 7 MHz linear probe. Tissue thickness was determined as the distance between the skin surface and the initial hypo-echoic structure (skin), the distance within the hypo-echoic structure (fat) and the distance between the fascias (muscle).

The skin and the subcutaneous tissues above, where the catheter entered the trapezius muscle were anaesthetized with a local injection (0.5 ml) of Xylocaine (20 mg/ml) without adrenaline, and care was taken not to anaesthetize the underlying muscle. Two commercially available microdialysis catheters (cut-off points of 20 and 100 kDa; CMA Microdialysis AB, Solna) were inserted with a catheter into the pars descendens of the trapezius muscle at half the distance between the processus spinosus of seventh cervical spine and the lateral end of the acromion. Typically, a brief involuntary contraction and change of resistance were perceived when the tip of the insertion needle of the catheter entered the fascia and muscle. The catheters were placed in the trapezius muscle parallel to the muscle fibers and perfused at 5 µl/min with a solution (perfusate) resembling the muscle interstitial fluid. The perfusion fluid consisted of Ringer acetate solution containing 3 mM glucose and 0.5 mM lactate and 0.3 µl/ml [^14^C]- Lactate (specific activity: 5.77 GBq/mmol; Amersham. Bucks. UK) as well as 0.3 µl/ml ^3^H_2_O (specific activity: 37 MBq/gram) were added to the perfusate used in the microdialysis catheters. This procedure allows the determination (if necessary, it turned out not to be the case) as to whether outlier values can be attributed to technical sampling errors, which would be reflected in abnormal *in vivo* relative recovery (RR) rates (calculated from 5 µl samples according to the internal reference method by Scheller and Kolb [29].

After the insertion of catheters, participants rested comfortably in an armchair for a 120 min period to allow the tissue to recover from possible changes in the interstitial environment induced by the operative procedure. After this period, participants continued to rest for a 20 minutes of baseline period. This was followed by a 20 min period of standardized repetitive low-force exercise performed on a pegboard (PEG). The experiment ended with a recovery period of 120 min during which participants rested. Immediately prior and after catheter insertion subjects were asked to rate their pain intensity. They continued to do so every 20 minutes throughout the experiment that lasted four hours. At 20 (i.e. beginning of trauma period), 120 (i.e. after recovery from the operative procedure), 140 (i.e. baseline), 160 (i.e. low-force exercise), 180, 200 and 220 min (i.e. recovery period) after the start of the experiment, microdialysate was collected in glass vials (CMA Microdialysis, Sweden). Each vial was weighted before and immediately after sampling order to confirm that sampling was working according to the perfusion rate set. All samples were controlled for visible signs of haemolysis that would result in the discarding of the sample. The samples were stored at 4°C throughout the experiment and then stored as aliquots −70°C until analysis.

In a review by Buczynski et.al. in 2010 important factors that can affect the sampling of extracellular NAE's and endocannbinoids were listed and recommendations were made as to the optimal sampling procedures [30]. Our study essentially follows the recommendations made by the review.

Participants were offered a standardized light meal at the 100 min time point. No food or beverages except for water was allowed otherwise during the experiment.

### Standardized low-force repetitive exercise

The low-force exercise consisted of a repetitive arm movement task that was performed unilaterally using the arm on the same side as the microdialysis catheter had been inserted in the trapezius [15]. The subjects moved short wooden sticks (11.8 g) back and forth between standardized positions 30 cm apart on a pegboard at a frequency of 1 Hz indicated by an electronic metronome (Korg Inc., Tokyo, Japan). The participants performed the exercise in a seated position with the pegboard placed 30 cm in front of them, measured from the elbow with the upper arm hanging vertically and the elbow in a 90° flexion. The exercise was supervised by qualified personnel (e.g. nurse or physiotherapist).

### Pain intensity ratings

The subjects were asked to rate their pain intensity on a numeric rating scale (NRS) with numbers (0–10; 0 = no pain and 10 = worst possible pain) provided along for guidance. All pain ratings concerned pain in the trapezius muscle of both the most painful side (subjects with pain) or the dominant side (pain free subjects) and the contralateral side.

### Analysis of NAE levels

On the day of analysis, 50 µl samples were dried by SpeedVacc. redissolved in methanol, vortexed and centrifuged. The supernatant was analysed by liquid chromatography - tandem mass spectrometry (LC-MS/MS). The EASY-nLC™ (Bruker Daltonics), a nano-flow HPLC system equipped with column 5 µm C18 (Nano Separations. 360 µm×75 µm i.d., 10 cm) coupled to a mass spectrometer Bruker Daltonics Ultra Performance High Capacity Ion Trap MS (HCT Ultra) with electrospray ionisation was used. The compounds were eluted isocratically (acetonitrile with 0.1% formic acid) at a flow rate of 0.0009 mL min^−1^. Online analysis of the NAEs was performed using the ion trap mass spectrometer HCT Ultra. The mass spectrometer was operated in the positive ion multiple reaction monitoring mode and the selected ion pairs used were 348.2/287.1. 300.2/283.1 and 328.3/311.1 m/z for AEA, PEA and SEA, respectively. The linearity of the measuring range was assessed with standard curves ranging from 0.01–20 nM (0.01; 0.012; 0.02; 0.025; 0.05; 0.1; 0.2; 1; 2; 5; 10; 20) in human muscle dialysate. Standard curves generated using linear regression.

### Statistics

Statistical analyses (D'Agostino and Pearson omnibus normality test, two-tailed Mann-Whitney U-test, Kruskal-Wallis test and Spearman's correlation test) were undertaken using the Graphpad Prism computer programme (GraphPad Software Inc, San Diego, CA, USA) and the PASW Statistics version 18 (IBM Corporation, Route 100 Somers, New York, USA).

## Results

### Measurement of NAEs in microdialysate samples from trapezius muscle

**Figure 1:**
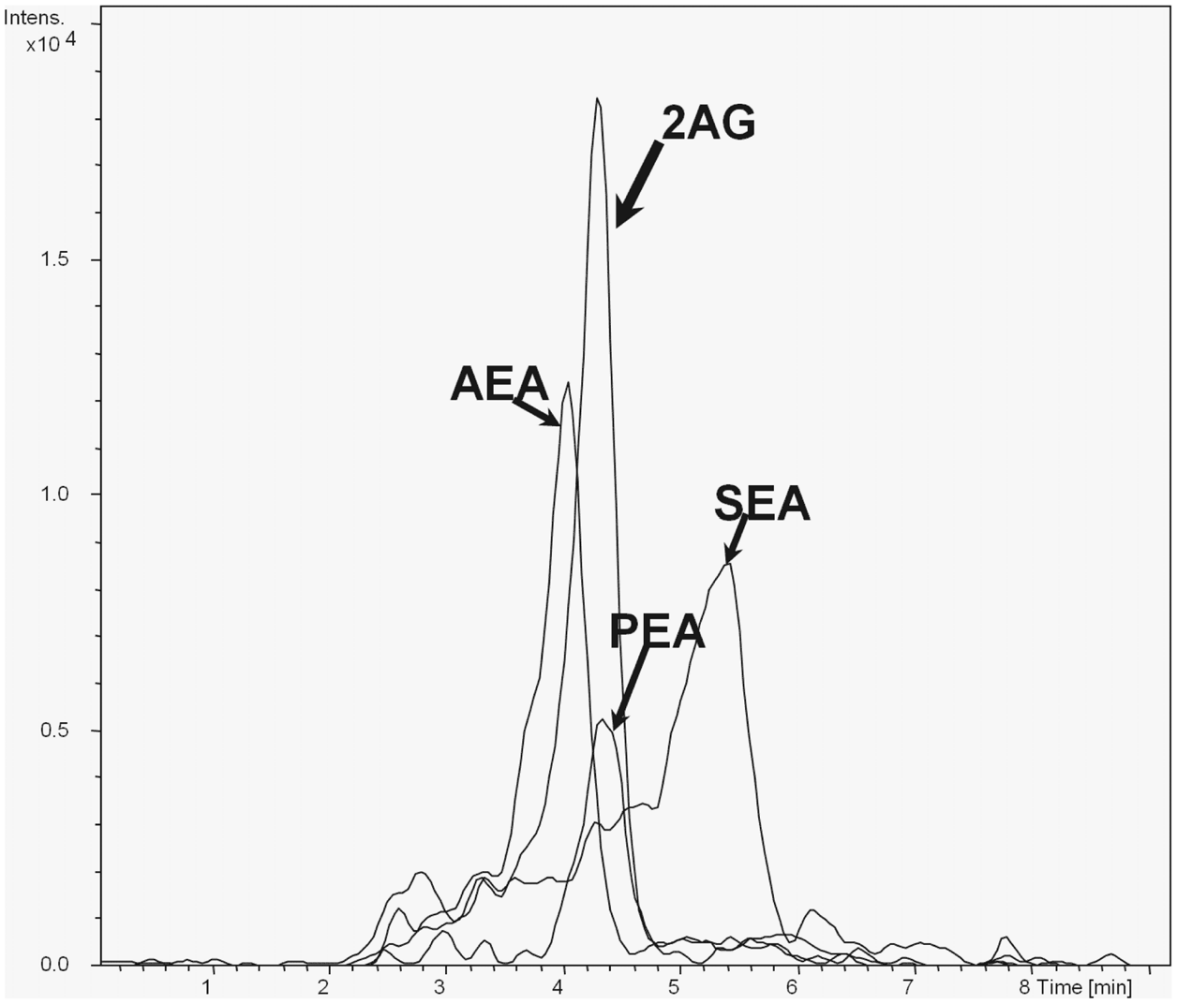
Representative MRM-chromatogram of reference standards. The injection volume was 4 µl for all of them (20 fM). The EASY-nLC™, a nano-flow HPLC system coupled to the mass spectrometer Ion Trap MS (HCT Ultra) was used as described in methods.

**Figure 2:**
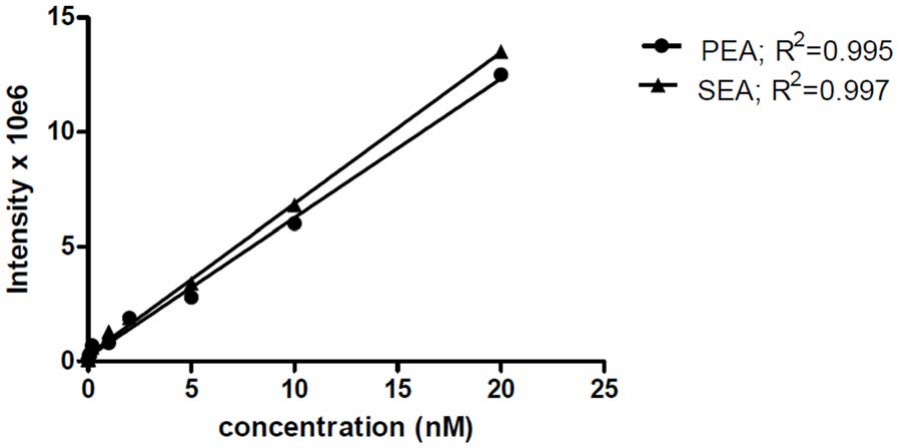
Example of standard response curves of PEA and SEA in human muscle dialysate.

A bioanalytical method utilizing nLC (liquid chromatography) MS/MS (tandem mass spectrometry) was used to measure NAEs and we were able to detect AEA, PEA, 2-AG, SEA, in a single chromatographic run (see **Fig. 1** for a representative chromatogram of reference standards). We could also separate these NAEs from three other NAEs: *N*-linolenoyl-, *N*-linoleoyl- and *N*- docosahexaenoyl - ethanolamines (data not shown). In initial pilot studies using pooled microdialysate samples from trapezius muscle of myalgia patients, however, only two NAEs could be robustly measured, PEA and SEA, and so we focussed upon these two in the main study. The linear measuring range for PEA and SEA was 0.05–20 nM in this study. **Figure 2** represents an example of standard response curves for PEA and SEA in muscle dialysate from women. The correlation coefficient of calibration curves (R^2^) in the diagrams were ≥0.9. The limit of detection with considering signal to noise ratio of 2 was 0.01 nM and the limit of quantitation was about 0.04 nM.

### Characterisation of levels of PEA and SEA in trapezius myalgia

**Figure 3:**
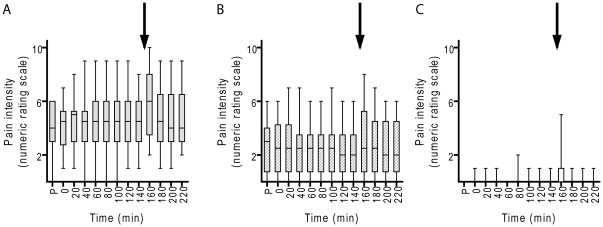
Pain intensity ratings for the myalgia patients and pain-free controls. Pain intensity ratings at the different time points (P = measurement prior to the surgery) for A, myalgia patients, most painful side; B, myalgia patients, contralateral side; C, pain-free controls, dominant side. Shown are box plots with the range of scores at each time point, n = 10–11. The arrow indicates when the standardized low-force repetitive exercise was undertaken.

Eleven patients with trapezius myalgia and eleven controls took part in the study. The pain scores (NRS) for the patients were relatively stable throughout the study. However, the median pain intensity increased significantly during the low force exercise in the patients with trapezius myalgia (baseline (140 min): 5 vs. exercise (160 min): 6; p = 0.028) but not in controls (baseline (140 min): 0 vs. exercise (160 min): 0; p = 0.102) (**Figure 3** **; Panel A**). The pain intensity of the contralateral side (i.e., clinically less painful) was generally lower throughout the experiment and did not show significant changes throughout the experiment in patients (**Figure 3** **; Panel B**). The median values for the control cases was 0 at all time points and the range was 0–2 except for the 160 min time point where one case reported a score of 5 on the dominant side (**Figure 3** **; Panel C**).

**Figure 4:**
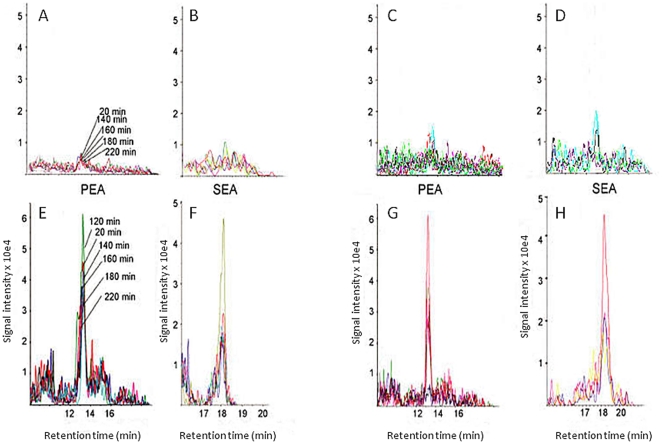
Examples of PEA and SEA chromatograms from dialysates from two controls (Panels A–D) and two cases with trapezius myalgia (Panels E–H). The analyses are shown in adjacent panels (i.e. A–B, C–D, E–F and G–H) for each subject.

In all subjects taken together there were no significant correlations between the concentrations of PEA and SEA and age or BMI. Higher concentrations of PEA and SEA were found in the trapezius muscle of pain subjects compared to pain free subjects. The difference between the groups was significant at all time points for both substances except for PEA at 140 min (**Table 2**). In the pain group there were no significant correlations between the concentrations of these substances and pain duration or pain intensity. There were no significant effects of sample time upon the observed values for NAE, either patients or control cases (P>0.2), suggesting that the interstitial levels of PEA and SEA were rather stable and were not affected by the period of standardized low-force repetitive exercise. Similar results were obtained when recovery of [^14^C] - lactate was used to standardize the samples (data not shown). Typical chromatograms for PEA and SEA in dialysate from two myalgic and two control subjects are shown in **Figure 4**.

**Table 2:** PEA and SEA concentrations (nM) in microdialysis samples from patients with trapezius myalgia and controls.

	Time of sample (min after start of experiment)					
	20)	120	140	160	180	220
	beginning of trauma period	after recovery from the operative procedure	baseline	low force exercise	recovery period	recovery period
**PEA**						
***Myalgia***	3.3 (0.42–11.9)	1.90 (0.58–4.10)	1.95 (0.10–8.60)	1.60 (0.60–6.60)	2.40 (0.92–6.80)	1.00 (0.40–4.50)
*n*	11	9	10	9	10	7
***Pain free subjects***	0.60 (0.10–2.5)	0.73 (0.50–2.50)	0.51 (0.30–3.70)	0.20 (0.10–1.8)	0.50 (0.10–1.2)	0.60 (0.10–0.90)
*n*	11	8	10	9	11	11
*P-value*	<0.001	0.011	0.15	<0.005	<0.001	0.018
**SEA**						
***Myalgia***	3.75 (1.4–16.5)	4.80 (0.65–20.0)	2.93 (0.63–18.0)	3.80 (0.20–13.0)	5.40 (0.96–20.0)	1.92 (0.45–12.0)
*n*	11	9	10	8	10	7
***Pain free subjects***	0.90 (0.10–2.00)	0.30 (0.10–1.70)	0.45 (0.10–2.1)	0.40 (0.10–2.4)	0.40 (0.10–1.8)	0.20 (0.05–2.00)
*n*	11	8	10	9	11	11
*P-value*	<0.001	0.002	0.003	0.018	<0.002	<0.01

Shown are median values with range in brackets. P values were determined by two-tailed Mann-Whitney U-test at each time point. For each series, there was no significant variation of the microdialysate concentrations over time (P>0.2, Kruskal-Wallis test). Non-parametric statistics were chosen since approximately half of the PEA data series and two of the SEA series (time points and pain conditions analysed separately) did not pass the D'Agostino and Pearson omnibus normality test.

## Discussion

The microdialysis technique has been used in a number of pain conditions in human, such as work-related chronic trapezius myalgia [15], [16], [31], [32], [33], [34], chronic whiplash associated disorders (WAD) with involvement of the trapezius [9], chronic myofascial pain [35] and fibromyalgia [36]. Most of these microdialysis studies report alterations in concentrations of algesic substances together with metabolic alterations in myalgic muscles. To date little is known about changes in the pain-inhibitory signaling molecules in chronic myalgia in humans. In the present study, we have developed a method allowing determination of NAEs in small samples of muscle dialysate and used the method to demonstrate that the levels of PEA and SEA are significantly elevated in women with chronic trapezius myalgia compared to healthy controls.

For detection of NAEs and/or endocannabinoids in biological samples, different analytical methods have been reported in the literature [37]. Most of the methods use a triple quadrupole tandem mass spectrometer and detect the typical fragment ion m/z: 62 which correspond to the protonated ethanolamine from NAEs. In this study, an alternative MRM (multiple reaction monitoring) method was developed with increased sensitivity and specificity with the long-term aim to detect NAEs from single dialysate samples using an ion-trap mass spectrometer (**Figure 1**). Measurement of such lipophilic molecules in microdialysis experiments is far from easy. Several factors such as composition and cut-off limit of the dialysate membrane, perfusate composition, perfusate flowrate, and collection time can effect the sampling of interstitial NAEs.

The concentration of these lipids in the dialysate samples will not represents the exact interstitial concentration in the trapezius muscle. However, this technique enables sampling of substances at the site of action. Also in this study we have used a membrane with a low cut-off (20 kDa) and this provides protein-free samples and prevents enzymatic degradation as soon as the lipids cross the dialysis membrane.

Recommendations have been made as to optimal sampling conditions [30]. Essentially following these recommendations, we have been able to detect PEA and SEA in microdialysis samples from trapezius muscle, whereas we were not able to detect 2-AG or AEA. It is possible that further optimization is required to detect unsaturated NAEs as opposed to the saturated PEA and SEA. However, in most tissues, AEA levels are an order of magnitude lower than those of PEA and SEA [38], and so it is unlikely that we would be able to detect this compound even under optimum conditions without dramatically increasing the size of our microdialysate samples. In consequence, we have elected to focus upon PEA (and SEA), since the small sample sizes allow for repeated measurements to assess the stability of the measured levels and to follow the outcome of an intervention, such as the standardized low-force repetitive exercise paradigm used here.

The present study demonstrates that levels of PEA and SEA are both increased in dialysate samples from women with chronic trapezius myalgia compared to healthy controls, and that this increase is rather stable over time and not obviously affected by the standardized low-force repetitive exercise work associated with a significant pain intensity increase. Hence, the elevated levels of PEA and SEA in chronic trapezius myalgia at rest were apparent, but these levels did not parallel the significant increase in pain during the work period. Thus, we could not show definite alterations in PEA and SEA linked to increased pain intensity during the work period. One explanation of this is that the levels of these substances are at maximum attainable in response to the pathological situation and cannot be further augmented by the increased pain. The lack of correlations between the concentrations of PEA and SEA and pain intensity and pain duration also give som support to our explanation. The lack of increases in PEA and SEA in controls could be due to the fact that the low force exercise did not elict pain in this group (**Figure 3** **; Panel C**). In future studies of the roles of PEA and SEA in relation to pain a design including provocation of pain both in healthy subjects and in patients with chronic pain is preferable. The number of subjects in this study were relatively low (11+11) which also limits the possibility to detect alterations in concentrations of PEA and SEA. Hence, in future studies it will be important to investigate if the increased levels of PEA and SEA represent a tonic situation or if also phasic aspects are present in chronic trapezius myalgia. Within such studies it is necessary to determine how NAEs react to acute muscle nociception/pain (e.g., using the intramuscular hypertonic saline model [39], [40]) in pain free controls. Only women were included in this study. In future studies it would be interesting to analyze these substances in men with chronic myalgia as well as healthy controls in order to investigate possible gender differences.

This new data with significant increases in PEA and SEA is of potential importance not only in diagnostic terms (currently history-taking and clinical examination are the only methods available for diagnosing chronic myalgia, and diagnostic biomarkers are greatly needed) but also in identifying potential novel therapies, given the analgesic properties of PEA [20], [21], [22], [23], [24], [41]. Less is known about SEA, although preliminary data from a model of experimental burn in rats have shown that SEA externally applied has an anti-inflammatory effect [42]. The large increase in the level of these compounds in myalgic muscles may reflect an attempt by the body to counteract noxious processes and activation of nociceptors as a result of the myalgia. If this is the case, then compounds preventing their breakdown of PEA and possibly SEA may be useful analgesics for this indication. PEA is metabolised by two different hydrolytic enzymes, fatty acid amide hydrolase (which also metabolises SEA) and *N*-acylethanolamine-hydrolyzing acid amidase. Recently, an inhibitor of *N*-acylethanolamine-hydrolyzing acid amidase has been described and shown to have anti-inflammatory properties [43]. Fatty acid amide hydrolase inhibition has been more extensively characterised in view of the ability of the enzyme to metabolise AEA, and inhibitors of this enzyme are currently under pharmaceutical development as analgesics (see [44]). Although more work is needed, the present study raises the possibility that such compounds may have utility in myalgia.

### Conclusion

There is a need for a better understanding of nociceptive and antinociceptive processes in chronic myalgia. The present study demonstrates that microdialysis in combination with mass spectrometry can be used for analysing *N*-acylethanolamines (NAEs) in human muscle tissue regularly over time. The significant group differences in the concentration of the pain-inhibitory signaling molecules PEA and SEA might fill an important gap in our knowledge of mechanisms in chronic myalgia in humans. In the long run an expanded understanding of anitinociceptive processes in the muscle may provide a base for ameliorating treatment and rehabilitation of chronic pain conditions.
